# Duration of Daytime Napping Is Related to Physical Fitness among Chinese University Students

**DOI:** 10.3390/ijerph192215250

**Published:** 2022-11-18

**Authors:** Lingfeng Kong, Yufei Cui, Qiang Gong

**Affiliations:** 1 Department of Physical Education, Hohai University, 1 Xikang Road, Nanjing 210098, China; 2 Department of Physical Education, Huaiyin Institute of Technology, Huaian 223003, China; 3 Department of Medicine and Science in Sports and Exercise, Graduate School of Medicine, Tohoku University, Sendai 9808575, Japan

**Keywords:** duration of napping, physical fitness, university students, cross-sectional study

## Abstract

Reportedly, daytime napping affects the physical fitness of athletes. However, results of these studies are conflicting, and may not be generalizable to all populations. Early adulthood is an important period linking adolescents and adults, during which building good physical fitness is crucial for their remaining lives. Thus, we investigated whether daytime napping duration is associated with physical fitness among Chinese university students. This study was based on an annual physical health examination for all university students and included 11,199 participants (6690 males; 4509 females). The daytime napping duration was assessed using a self-report questionnaire. Physical fitness was measured with a 50 m sprint; 1000 m (for males) and 800 m (for females) runs; standing long jump, sit-and-reach, pull-up (for males), and sit-up (for females) tests; and vital capacity. The adjusted association was evaluated using analysis of covariance. Of the participants, 86% napped regularly. After covariate adjustment was performed, significant V-shaped associations were observed between the daytime napping duration and the 50 m sprint and 800 m run results in males and females. Inverted V-shaped associations were observed between the daytime napping duration and the sit-and-reach, standing long jump, and pull-up test performances and vital capacity in males and between the daytime napping duration and the standing long jump test performance in females. Daytime napping for <30 min may have beneficial effects on physical fitness among university students.

## 1. Introduction

In recent years, China has made considerable national efforts to promote work and research related to physical health. Although physical health indicators, such as height and weight, among Chinese adults improved from 2014 to 2020, physical fitness parameters, such as strength, flexibility, and agility, continue to decline, with percentages of 0.5, 3.8, and 1.8, respectively [1]. Maintaining good physical fitness is crucial for maintaining good overall health, as a decline in physical fitness is associated with negative health effects, such as functional impairment [2], poor bone health [3], disability [4], and poor mental health [5]. Early adulthood is an important period for habit formation [6], and developing good physical fitness during this period has the potential to have far-reaching consequences [7]. A low level of physical fitness is significantly correlated with the risk of all-cause death after a few years [8]. However, most people in this age group are college or university students. The pressure of independent living and busy schoolwork may result in lack of time for physical activity [9], which may lead to the physical fitness status remaining stagnant or even declining. In addition to the direct effects of physical activity, other approaches have been linked to physical enhancement, such as nutrition [10], dietary habits [11], and sleep habits [12].

Daytime napping is an efficient way to overcome nighttime sleep deprivation and fatigue [13,14]. Although daytime napping is related to many health outcomes, such as hypertension [15], diabetes [16] and mental disorders [17], the findings of previous studies on the effects of daytime napping are inconsistent. For example, a study indicated that a longer nap is associated with an increased risk of hypertension [15]. A J-curve relationship has been reported between the napping duration and risk of diabetes or metabolic syndrome [16]. An epidemiological study on the population in China demonstrated that a short nap duration demonstrates protective effects against symptoms of depression [17]. Daytime napping can help people recover from fatigue and improve their mental health. Thus, daytime napping may also be related to physical fitness because factors such as fatigue and mental health are correlated with physical fitness [18,19]. Studies have investigated the relationship between daytime napping and physical fitness in athletes. Some studies have indicated that a shorter napping duration is better for physical fitness [20,21], whereas others advocate longer napping durations [22,23]. However, this association has not been examined in young adults. We considered that young adults may achieve physical recovery through napping but that longer daytime napping consumes the time that may be allocated to physical activity or exercise, which may cause a decline in physical fitness. Therefore, there must be a reasonable range of napping duration for achieving better physical fitness in young adults. The aim of the present study was to investigate whether the daytime napping duration is associated with physical fitness and determine the most appropriate daytime napping duration among university students.

## 2. Materials and Methods

### 2.1. Participants

**Figure 1:**
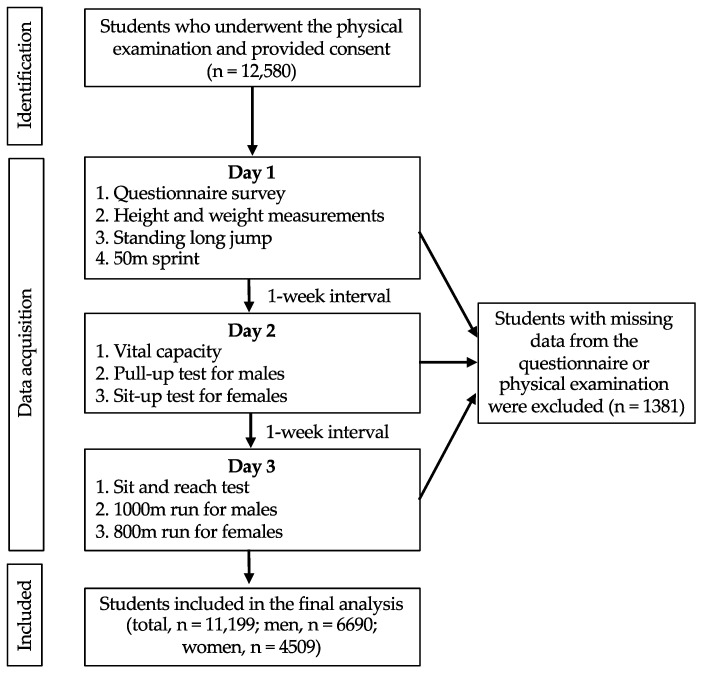
Flowchart of the sample selection process.

We recruited participants through an annual physical health examination of university students at Huaiyin Institute of Technology in Jiangsu, China, between September 2018 and October 2018. All students must have participated in the examination, and those who satisfied our study inclusion criteria were invited to participate in the study. Students with physical disabilities, cardiovascular diseases, respiratory diseases, or special reasons or circumstances that prevented them from participating were excluded before the physical examinations were performed. The physical health examinations were performed at the sports facilities by 40 experienced instructors working at the university. We administered a questionnaire under the guidance of the instructors before the examinations were performed. All of the participants voluntarily joined the study, and written consent was obtained from those willing to participate in the study. This study was approved by the ethics committee of Huaiyin Institute of Technology. A total of 12,580 students aged 19.6 ± 1.4 years agreed to participate in the study. We excluded participants whose questionnaire data (*n* = 543) or physical fitness data (*n* = 838) were missing. The final study population included 11,199 participants (6690 male students and 4509 female students). The sample selection process is shown in Figure 1.

### 2.2. Physical Fitness

All measurements of physical fitness were based on the National Student Physical Health Standard (2014 revision) [24]. The following physical fitness tests were conducted using the criteria for university students, and an 800 m jogging warm-up was performed before the physical examination, and stretching warm-ups were performed before each item of the physical examination. All tests were conducted and recorded by professional instructors trained before implementing the physical fitness examination.

50 m sprint: The 50 m sprint was performed on an outdoor standard track surface. The students were encouraged to attempt their best to run until they touched the finish line. One familiarization run was performed before the test. The run time was recorded in seconds. 

1000 m and 800 m runs: The 1000 m run was used for the male participants, whereas the 800 m run was used for the female participants. The running tests were performed on a standard track surface. The total time required to complete the distance was recorded in minutes and seconds. 

Standing long jump test: This test was performed on an indoor synthetic-surface track, and the results were recorded within an accuracy of 1 cm. The distance was measured from the takeoff line to the point where the back of the heel (nearest to the takeoff line) landed on the ground. A familiarization run was performed before the test, and the test was measured three times for each student who completed the test three times. The highest value of the three test runs was included in the analysis.

Sit-and-reach test: The test was conducted indoors with the shoes removed. Students sat on a yoga mat with their legs stretched out straight ahead. Both knees were locked and pressed flat onto the floor. With the palms facing downwards and the hands side-by-side, the students slowly reached forward along the measuring line as far as possible. Each student underwent the test once, and the distance (in cm) was then recorded. 

Pull-up test: The male students underwent this test. The student grasped the overhead bar using an overhand grip with the arms fully extended, raised the body until the chin cleared the top of the bar, and then lowered again to a position with the arms fully extended. The time to completion was recorded.

Sit-up test: The female students underwent this test. The students were instructed to lay on a gym mat with their knees bent at approximately right angles and with their feet flat on the ground. Their hands were placed behind their heads. A partner anchored their feet. After a start instruction was communicated, the participants squeezed their stomachs and allowed their backs to raise high enough for their elbow to touch the tops of their knees, and they then returned to the starting position. The number of sit-ups completed in 1 min was recorded.

Vital capacity: This parameter was measured using a spirometer (JH-1663, Jihao Electron Co., Changzhou, China), with the participants in a standing position. Starting from the total lung capacity, the participants were instructed to perform a maximal slow expiration through a mouthpiece connected to the spirometer. The results were recorded as the vital capacity (mL) / body weight (kg).

### 2.3. Daytime Napping

The duration of daytime napping was ascertained using a single item in the questionnaire. This item asked the students to report their typical napping duration on an average day in hours and minutes. The students often set alarms to wake up. Most alarms were set for a predetermined time, such as 30, 60, or 90 min. Considering this, the daytime napping duration was categorized as none, <30 min, 31–60 min, 61–90 min, and >90 min.

### 2.4. Physical Activity

Physical activity (PA) was assessed using the International Physical Activity Questionnaire Short form (IPAQ-short) [25], and metabolic equivalents (METs) were calculated. Then, the total physical activity was calculated as: METs × h/week. Physical activity in the present study was used as a continuous variable.

### 2.5. Covariates

The following variables were considered to be potential confounders: grade (freshman, sophomore, junior, or senior), race (Han or minority), body mass index (BMI) in kg/m^2^ (continuous), living expenses (low, medium, or high), living status (dormitory or other), smoking status (smoker or nonsmoker), alcohol consumption status (nondrinker, drinking 1–2 times/week, or drinking >2 times/week), nighttime sleep duration (7–8 h/day or other), and depressive symptoms assessed using the Zung Self-Rating Depression Scale (with or without depression) [26].

### 2.6. Statistical Analysis

The statistical analyses were performed using Statistical Package for Social Sciences for Mac (SPSS for Mac, version 24.0; IBM Corporation, Armonk, NY, USA). Bivariate correlation analyses were used to evaluate the unadjusted associations in the participants’ characteristics. Group differences were examined using analysis of variance (ANOVA) for continuous variables and using the χ^2^ test for categorical variables. Analysis of covariance (ANCOVA) was performed to examine whether participants with long and short daytime napping durations differed in terms of physical fitness after adjusting for relevant covariates. The items of physical fitness were used as dependent variables (continuous variable), and the duration of daytime napping was used as the independent variable (categorical variable). Linear and quadratic trends were considered for the association between the daytime napping duration and physical fitness. Log transformation was performed to data that did not follow normal distribution. The test items for men and women were different; thus, we analyzed both sexes separately. Associations were considered significant when the *p*-value was <0.05.

## 3. Results

Table 1 shows the general characteristics of the male and female students according to the daytime napping duration. The proportions of students who were sophomores, were juniors, had high living expenses, were drinking 1–2 times per week, and had depressive symptoms and physical activity levels were higher across the duration of daytime napping categories in the male participants. The proportions of students who were freshmen, had low living expenses, were nonsmokers, were nondrinkers, and were sleeping 7–8 h per night were lower across the duration of daytime napping categories in the male participants. The proportions of students who were juniors, had medium or high living expenses, and had depressive symptoms were higher across the duration of daytime napping categories in the female participants. In contrast, the proportions of students who were seniors, had low living expenses, and were nondrinkers were lower across the duration of daytime napping categories.

The adjusted association between the duration of daytime napping and physical fitness in the male participants is shown in Table 2. There were few significant associations in the unadjusted models (crude model). However, these results changed after adjusting for the confounding factors. In Model 2 (final adjusted model), there was a positive linear association observed between the duration of daytime napping and performance in the 1000 m run (*p* for linear trend = 0.030). Compared with the linear association, V-shaped associations were also observed between the duration of daytime napping and performances in the 50 m sprint and 1000 m run (*p* for quadratic trend = 0.031 and *p* for quadratic trend < 0.001, respectively), whereas inverted V-shaped associations were observed between the duration of daytime napping and performances in the standing long jump, sit-and-reach, and pull-up tests and vital capacity (*p* for quadratic trend < 0.001, *p* for quadratic trend = 0.001, *p* for quadratic trend = <0.001, and *p* for quadratic trend = 0.029, respectively). In the male students, a napping duration of <30 min was associated with the best score for physical fitness.

Table 3 shows the adjusted association between the duration of daytime napping and physical fitness in the female participants. Similar to the male participants, significant V-shaped associations were observed between the daytime napping duration and performances in the 50 m sprint and 800 m run (both *p* for quadratic trend < 0.001). An inverted V-shaped association was observed between the daytime napping duration and performance in the standing long jump test (*p* for quadratic trend = 0.007). In contrast, a longer daytime napping duration was significantly associated with a poor sit-and-reach performance (*p* for linear trend = 0.002). However, there was no significant association between the daytime napping duration and the sit-up test performance or vital capacity.

## 4. Discussion

This study revealed an association between the duration of daytime napping and physical fitness among university students. The results suggest that a daytime napping duration of <30 min has the potential to enhance physical fitness among university students. The confounding factors in the full-adjustment model strengthened this inverse association.

More than 86% of university students in our study had napping habits, which is similar to that observed in a Spanish [27] and an American [28] study that demonstrated that >80% of university students have napping habits, whereas it is higher than that observed in other studies. For example, the prevalence of napping among Australian university and Mexican college students is 54.6% [29] and 34% [30], respectively.

The relationship between napping and physical fitness is well-investigated in athletes, but not in the general population. Some of these findings of previous studies are consistent with our findings. A study confirmed the effects of a 30 min napping duration on sprinting performance and reported that an improved sprinting performance was observed in the napping group [20]. Furthermore, the maximum speed of sprinting significantly increases after a 25 min nap [21]. Another study that investigated the effect of napping on jumping performance demonstrated that 30 min of napping increased the countermovement jump and squat-jump performances [31]. A 30 min nap also has a greater beneficial effect on the 3000 m run performance than that in a control group [32]. Some studies have reported results that are completely or partially inconsistent with those of our study. For example, a study indicated a significant increase in physical fitness after a 45 min nap, but no significant effect was observed in that after a 25 min nap [14]. In addition, a study investigated the effects of 90 min and 20 min napping durations on sprinting performance. The results demonstrated that both nap durations enhanced the maximum power but that increases in the minimum power and mean power were observed in only those with the long nap duration (90 min) [22]. The notion of a longer napping duration improving exercise performance was also supported by the study by Boukhris et al., which indicated that the anaerobic capacity of individuals was greater with a 90 min nap than with a 40 min nap [23]. To summarize, previous studies examined one or two lengths of daytime naps in a single study. Thus, it may be difficult to determine whether longer or shorter napping durations are better for physical fitness. In addition, the participants in these studies were athletes. It could also be considered that a longer napping duration is better for athletes because they must recover from training to improve their performance in sports through sleep [33]. However, these points are the main differences between previous studies and the present study. In contrast, the present study demonstrated a linear association between the napping duration and sit-and-reach test performance in female participants. This is different from other results that demonstrated a V-shaped association between these variables. However, the results of sit and reach among participants in the “none” and <30 min napping groups were 17.22 and 17.39, respectively. Although the quadratic trend was not significant, students with a napping duration of <30 min scored slightly higher on this test. This finding is partially consistent with our other finding that a long duration of daytime napping is associated with a lower level of physical fitness.

To our knowledge, this is the first study to examine the association between the duration of daytime napping and physical fitness (including running capacity, strength, and flexibility) in university students. The present study strengthens the evidence for the association between napping duration and physical fitness and expands its association to the young-adult population. The mechanism underlying the association between daytime napping and physical fitness can be demonstrated in several ways. First, daytime napping can enhance antioxidant defense mechanisms by reducing sleep loss-induced muscle and oxidative damage [22]. In addition, it has been reported that napping normalizes sleep loss-induced biochemical disruption [34]. Thus, daytime napping could be related with physical fitness due to sleep, and oxidant status is an important factor for physical fitness [12,35]. However, the explanation that links daytime napping and physical fitness in university students is different from that in athletes. The present study demonstrated that < 30 min of daytime napping is the optimal napping duration from five napping duration categories of physical fitness in university students. Considering that it is unnecessary for ordinary students to physically recover through long naps, a long napping duration may consume their time that would have been otherwise allocated to studying or exercising, which directly or indirectly affects the physical fitness of students. We also hypothesized that long naps may cause a reduction in daily physical activity, which consequently leads to weaker physical fitness. However, the results did not change after adjusting for physical activity. Thus, in our study, physical activity was independent of the association between napping duration and physical fitness.

There are some limitations of the present study that must be mentioned. First, the current study lacked an objective measurement of the daytime napping duration. This aspect should be considered in future studies. Second, because of the cross-sectional nature of the study, causality cannot be addressed. We cannot conclude whether the duration of daytime napping influences physical fitness or whether physical fitness leads people to having daytime naps at one time point. Third, although the instructors were trained before the physical examination and survey administration, measurement bias between the instructors may still exist. Fourth, variables other than physical fitness were assessed by self-reporting, which may make these variables be subject to recall bias. Fifth, we evaluated alcohol drinking by the frequency of drinking, which does not represent the amount of alcohol consumed, and this may have confounding effects on the results. Sixth, although we did not aim to confirm differences between sexes, we could not obtain a representative result between running ability and upper muscle strength due to the running distances being different between the tests of the two sexes (1000 m for males and 800 m for females). Likewise, pull-ups (for males) use the latissimus dorsi and arm muscles, but sit-ups (for females) use the abdominal muscles. Finally, although adjustments for several potential confounding factors were considered, the possibility that other covariates may have mediated the association between the duration of daytime napping and physical fitness cannot be excluded.

## 5. Conclusions

The present study examined the association between the daytime napping duration and physical fitness. The results revealed that university students with <30 min of daytime napping had better physical fitness than those with more than 30 min of daytime napping. Although this association was not observed for several items of physical fitness among the female participants, the study findings also support previously published data in studies analyzing the napping duration and physical fitness. In the future, longitudinal studies or randomized trials should be conducted to establish causality. Although some previous findings suggested that a longer napping duration is beneficial for athletes, for ordinary university students, a shorter napping duration may be a better choice for maintaining optimal physical fitness. The present results also provide important information for the fields of preventative medicine and health education.

## Figures and Tables

**Table 1:** Characteristics of male and female participants according to the duration of daytime napping.

	Duration of Daytime Napping (Minutes)					Effect Size ^a^	Trend *p* ^b^
	None	Less than 30	31–60	61–90	>90		
Male participants							
n	1038	798	2072	866	1917		
BMI (kg/m^2^) ^c^	22.0 (21.8, 22.2) ^d^	22.1 (21.8, 22.3)	22.1 (22.0, 22.3)	22.0 (21.8, 22.3)	21.9 (21.8, 22.1)	0.001	0.589
Grade (%)							
Freshman	26.0	37.1	32.9	30.9	22.5	0.113	<0.001
Sophomore	26.3	28.3	26.4	31.9	30.6	0.048	0.003
Junior	28.2	20.6	24.1	23.6	31.0	0.083	0.001
Senior	19.5	14.0	16.5	13.6	15.9	0.048	0.049
Minority race (%)	3.9	3.6	3.2	4.8	4.3	0.030	0.243
Living expenses (%)							
Low	39.7	45.9	40.5	41.6	36.3	0.060	0.002
Medium	50.4	47.4	51.1	49.5	52.5	0.031	0.111
High	9.9	6.8	8.4	8.9	11.3	0.051	0.018
Living status (dormitory; %)	99	98.7	99.4	98.8	98.9	0.025	0.575
Nonsmoker (%)	92.2	93.9	93.2	91.2	88.2	0.078	<0.001
Drinking status (%)							
Nondrinker	71.7	71.4	71.8	65.7	63.5	0.081	<0.001
Drink 1–2 times/week	24.8	26.7	25	31.8	32.4	0.078	<0.001
Drink >2 times/week	3.6	1.9	3.2	2.5	4.1	0.040	0.149
PA (METs hour/week)	51.6 (48.8, 54.4)	46.3 (43.1, 49.5)	49.4 (47.4, 51.4)	51.6 (48.5, 54.6)	54.8 (52.8, 56.9)	0.004	0.005
Sleep duration (7–8 h/day; %)	50.9	55.8	53.5	52.8	49.0	0.053	0.043
Depressive symptoms (%)	11.1	9.4	9.1	12	15.8	0.086	<0.001
Female participants							
n	526	616	1373	769	1225		
BMI (kg/m^2^)	20.5 (20.2, 20.7)	20.4 (20.2, 20.6)	20.4 (20.2, 20.5)	20.4 (20.2, 20.6)	20.4 (20.2, 20.5)	<0.001	0.699
Grade (%)							
Freshman	24.9	31.5	36.1	32.5	27.8	0.084	0.789
Sophomore	35.4	33.6	30.7	30.6	30.6	0.037	0.042
Junior	29.1	20.9	25.1	29.4	35.7	0.112	<0.001
Senior	10.6	14	8.1	7.3	6	0.093	<0.001
Minority race (%)	4.2	6.7	5.2	5.5	6.8	0.038	0.104
Living expenses (%)							
Low	36.3	45.3	35	38.5	27.2	0.121	<0.001
Medium	52.7	46.8	54.6	52	57.9	0.070	0.001
High	11	8.0	10.3	9.5	14.9	0.077	<0.001
Living status (dormitory; %)	99.2	99	99.3	98.8	99.2	0.020	0.812
Nonsmoker (%)	99.8	99.8	99.6	99.9	99	0.049	0.014
Drinking status (%)							
Nondrinker	96.2	95.9	95.8	96.9	93.7	0.055	0.02
Drink 1–2 times/week	3.2	3.9	4.2	3	5.5	0.045	0.053
Drink >2 times/week	0.6	0.2	0.1	0.1	0.8	0.053	0.107
PA (METs hour/week)	53.3 (49.3, 57.3)	47.7 (44.0, 51.3)	47.6 (45.1, 50.1)	51.1 (47.9, 54.4)	52.6 (49.9, 55.2)	0.003	0.709
Sleep duration (7–8 h/day; %)	57.6	59.9	61	62.7	53.9	0.074	0.052
Depressive symptoms (%)	13.7	10.1	10.1	9.1	16.6	0.093	0.004

^a^ Showed as η^2^ for ANOVA and *V* for χ^2^ test, and >0.1 was considered small effect size. ^b^ Obtained using ANOVA for continuous variables and χ^2^ test for proportional variables. ^c^ BMI: body mass index. PA: physical activity. ^d^ Mean; 95% CI in parentheses (all such values).

**Table 2:** Adjusted relationship between the duration of daytime napping and physical fitness in 6691 males.

	Duration of Daytime Napping (Minutes)						
	None	Less than 30	31–60	61–90	>90	*p* for Linear Trend ^b^	*p* for Quadratic Trend ^b^
n	1038	798	2071	866	1917		
50 m sprint (second)							
Crude	7.38 ^a^ (7.35, 7.41)	7.36 (7.33, 7.40)	7.39 (7.37, 7.41)	7.39 (7.36, 7.42)	7.38 (7.36, 7.40)	0.620	0.903
Model 1 ^c^	7.39 (7.36, 7.41)	7.35 (7.32, 7.38)	7.38 (7.36, 7.40)	7.39 (7.36, 7.42)	7.39 (7.37, 7.41)	0.259	0.178
Model 2 ^d^	7.38 (7.36, 7.41)	7.34 (7.31, 7.37)	7.37 (7.36, 7.39)	7.39 (7.36, 7.42)	7.40 (7.38, 7.42)	0.051	0.031
1000 m run (second)							
Crude	256.5 (255.0, 258.0)	251.4 (249.7, 253.2)	254.6 (253.5, 255.6)	254.7 (253.0, 256.3)	255.9 (254.8, 257.0)	0.374	<0.001
Model 1 ^c^	256.6 (255.2, 257.9)	251.3 (249.8, 252.9)	254.2 (253.3, 255.2)	254.8 (253.3, 256.3)	256.2 (255.2, 257.2)	0.173	<0.001
Model 2 ^d^	256.5 (255.1, 257.8)	250.9 (249.3, 252.4)	254.0 (253.0, 255.0)	254.9 (253.5, 256.4)	256.7 (255.8, 257.7)	0.030	<0.001
Standing long jump (cm)							
Crude	223.0 (228.9, 231.1)	231.2 (229.9, 232.4)	230.4 (229.7, 231.2)	230.6 (229.4, 231.8)	229.8 (229.0, 230.6)	0.591	0.094
Model 1 ^c^	229.6 (228.6, 230.7)	231.7 (230.5, 232.9)	230.7 (230.0, 231.5)	230.8 (229.6, 231.9)	229.4 (228.6, 230.2)	0.374	0.001
Model 2 ^d^	229.7 (228.6, 230.7)	231.9 (230.7, 233.1)	230.9 (230.1, 231.6)	230.7 (229.6, 231.9)	229.1 (228.4, 229.9)	0.143	<0.001
Sit and reach test (cm)							
Crude	11.85 (11.50, 12.19)	12.72 (12.32, 13.12)	12.12 (11.87, 12.36)	12.19 (11.81, 12.58)	11.73 (11.48, 11.99)	0.154	0.001
Model 1 ^c^	11.79 (11.45, 12.14)	12.73 (12.33, 13.13)	12.10 (11.85, 12.34)	12.23 (11.85, 12.61)	11.76 (11.51, 12.02)	0.281	<0.001
Model 2 ^d^	11.80 (11.45, 12.15)	12.71 (12.31, 13.10)	12.10 (11.85, 12.34)	12.22 (11.84, 12.60)	11.78 (11.52, 12.04)	0.309	0.001
Pull-up (times/minute)							
Crude	0.53 (0.50, 0.55) ^e^	0.58 (0.55, 0.60)	0.55 (0.53, 0.57)	0.53 (0.50, 0.56)	0.54 (0.52, 0.56)	0.632	0.093
Model 1 ^c^	0.52 (0.50, 0.54)	0.59 (0.56, 0.61)	0.56 (0.54, 0.57)	0.53 (0.51, 0.56)	0.54 (0.52, 0.56)	0.616	0.001
Model 2 ^d^	0.53 (0.52, 0.55)	0.59 (0.56, 0.61)	0.56 (0.54, 0.57)	0.53 (0.51, 0.56)	0.53 (0.52, 0.55)	0.379	<0.001
Vital capacity (mL/kg)							
Crude	64.25 (63.58, 64.92)	64.73 (64.97, 65.50)	64.30 (63.82, 64.77)	64.06 (63.32, 64.79)	64.92 (64.42, 65.41)	0.518	0.394
Model 1 ^c^	63.99 (63.49, 64.50)	65.19 (64.61, 65.76)	64.63 (64.27, 64.98)	64.16 (63.61, 64.72)	64.46 (64.09, 64.83)	0.911	0.044
Model 2 ^d^	63.98 (63.48, 64.48)	65.24 (64.66, 65.82)	64.63 (64.28, 64.99)	64.17 (63.62, 64.72)	64.43 (64.06, 64.81)	0.827	0.029

^a^ Variables are expressed as estimated geometrics means (95% CI). ^b^ Obtained using ANCOVA. ^c^ Adjusted for grade, body mass index, and race. ^d^ Further adjusted for living expenses, physical activity, living status, smoking and drinking habits, depressive symptoms, and sleep duration. ^e^ Variables were log-transformed for normal distribution.

**Table 3:** Adjusted relationship between the duration of daytime napping and physical fitness in 4509 females.

	Duration of Daytime Napping (Minutes)						
	None	Less than 30	31–60	61–90	>90	*p* for Linear Trend ^b^	*p* for Quadratic Trend ^b^
n	526	616	1373	769	1225		
50 m sprint (second)							
Crude	9.16 ^a^ (9.11, 9.21)	9.07 (9.02, 9.11)	9.10 (9.07, 9.13)	9.10 (9.06, 9.14)	9.15 (9.11, 9.18)	0.976	0.001
Model 1 ^c^	9.16 (9.12, 9.21)	9.06 (9.01, 9.10)	9.09 (9.06, 9.12)	9.10 (9.06, 9.13)	9.16 (9.13, 9.19)	0.720	<0.001
Model 2 ^d^	9.16 (9.12, 9.21)	9.06 (9.02, 9.10)	9.10 (9.07, 9.12)	9.10 (9.06, 9.14)	9.16 (9.12, 9.19)	0.752	<0.001
800 m run (second)							
Crude	244.0 (242.3, 245.6)	241.6 (240.1, 243.1)	241.7 (240.6, 242.7)	242.0 (240.6, 243.4)	245.5 (244.4, 246.5)	0.134	<0.001
Model 1 ^c^	243.7 (242.2, 245.3)	241.2 (239.7, 242.6)	241.7 (240.7, 242.6)	242.0 (240.7, 243.3)	245.8 (244.7, 246.8)	0.025	<0.001
Model 2 ^d^	243.8 (242.2, 245.4)	241.3 (239.8, 242.8)	241.6 (240.7, 242.6)	242.1 (240.8, 243.4)	245.6 (244.6, 246.7)	0.041	<0.001
Standing long jump (cm)							
Crude	170.6 (169.3, 171.9)	173.0 (171.8, 174.2)	171.7 (170.9, 172.5)	170.8 (169.8, 171.9)	171.0 (170.1, 171.8)	0.432	0.035
Model 1 ^c^	170.4 (169.2, 171.7)	172.9 (171.8, 174.1)	171.9 (171.1, 172.6)	170.9 (169.9, 172.0)	170.8 (169.9, 171.6)	0.421	0.006
Model 2 ^d^	170.4 (169.2, 171.7)	172.9 (171.7, 174.0)	171.9 (171.1, 172.7)	170.9 (169.8, 171.9)	170.8 (170.0, 171.6)	0.455	0.007
Sit and reach test (cm)							
Crude	17.24 (16.78, 17.70)	17.45 (17.03, 17.87)	16.97 (16.69, 17.26)	16.82 (16.44, 17.20)	16.53 (16.23, 16.83)	0.001	0.337
Model 1 ^c^	17.23 (16.78, 17.69)	17.42 (17.00, 17.84)	16.99 (16.71, 17.27)	16.83 (16.45, 17.20)	16.53 (16.23, 16.83)	0.001	0.303
Model 2 ^d^	17.22 (16.77, 17.68)	17.39 (16.97, 17.82)	16.99 (16.70, 17.27)	16.80 (16.42, 17.18)	16.56 (16.26, 16.87)	0.002	0.383
Sit-up (times/minute)							
Crude	32.49 (31.86, 33.13)	32.69 (32.10, 33.28)	32.75 (32.36, 33.14)	31.67 (31.14, 32.19)	32.60 (32.19, 33.02)	0.362	0.724
Model 1 ^c^	32.41 (31.79, 33.03)	32.65 (32.08, 33.23)	32.84 (32.46, 33.22)	31.72 (31.20, 32.23)	32.53 (32.12, 32.93)	0.406	0.852
Model 2 ^d^	32.43 (31.81, 33.05)	32.77 (32.20, 33.35)	32.85 (32.46, 33.23)	31.79 (31.28, 32.30)	32.40 (31.99, 32.81)	0.218	0.517
Vital capacity (mL/kg)							
Crude	55.21 (54.35, 56.08)	55.02 (54.22, 55.82)	54.79 (54.25, 55.32)	54.64 (53.92, 55.36)	54.89 (54.32, 55.45)	0.385	0.458
Model 1 ^c^	54.95 (54.22, 55.67)	54.97 (54.30, 55.64)	54.99 (54.55, 55.44)	54.78 (54.18, 55.38)	54.70 (54.23, 55.18)	0.499	0.691
Model 2 ^d^	54.94 (54.22, 55.67)	55.06 (54.39, 55.73)	55.01 (54.57, 55.46)	54.80 (54.20, 55.40)	54.62 (54.15, 55.10)	0.367	0.494

^a^ Variables are expressed as estimated geometrics means (95% CI). ^b^ Obtained using ANCOVA. ^c^ Adjusted for grade, body mass index, and race. ^d^ Further adjusted for living expenses, physical activity, living status, smoking and drinking habits, depressive symptoms, and sleep duration.

## Data Availability

The datasets used and analyzed during the current study are available from the corresponding author on reasonable request.
